# Development of Innovative Online Modularized Device for Turbidity Monitoring

**DOI:** 10.3390/s23063073

**Published:** 2023-03-13

**Authors:** Chen-Hua Chu, Yu-Xuan Lin, Chun-Kuo Liu, Mei-Chun Lai

**Affiliations:** 1 Green Energy and Environment Research Laboratories, Industrial Technology Research Institute, Hsinchu 310, Taiwan; 2 Center for Measurement Standards, Industrial Technology Research Institute, Hsinchu 310, Taiwan

**Keywords:** turbidity, online monitoring, water-quality

## Abstract

Given progress in water-quality analytical technology and the emergence of the Internet of Things (IoT) in recent years, compact and durable automated water-quality monitoring devices have substantial market potential. Due to susceptibility to the influence of interfering substances, which lowers measurement accuracy, existing automated online monitoring devices for turbidity, a key indicator of a natural water body, feature a single light source and are thus insufficient for more complicated water-quality measurement. The newly developed modularized water-quality monitoring device boasts dual light sources (VIS/NIR), capable of measuring the intensity of scattering, transmission, and reference light at the same time. Coupled with a water-quality prediction model, it can attain a good estimate for continuing monitoring of tap water (<2 NTU, error < 0.16 NTU, relative error < 19.6%) and environmental water samples (<400 NTU, error < 3.86 NTU, relative error < 2.3%). This indicates the optical module can both monitor water quality in low turbidity and provide water-treatment information alerts in high turbidity, thereby materializing automated water-quality monitoring.

## 1. Introduction

As an indicator of a water body’s appearance, turbidity refers to a water body containing such suspended substances as clay, mud, organic and inorganic colloid, or plankton, which make the water appear murky, due to the effect of light reflection or refraction; the higher the intensity of the scattered or attenuated light, the higher the value of turbidity [1,2,3,4]. Turbidity differs from suspended solids (SS) or color, with the former referring to water-insolvable materials incapable of passing through filter paper whose measurement unit is mg/L and the latter referring to water-body color after removal of suspended substances. Although many particles per se causing turbidity are harmless, they may be attached by germs and shield the latter from the effect of disinfectant. Therefore, higher-than-standard turbidity indicates problems with water cleanness and water-supply pipes, producing the need for a better, real-time in-pipe water quality monitoring system to be deployed in the water distribution network and at consumer sites to provide immediate improvement to safeguard drinkers’ health [5,6,7].

**Figure 1:**
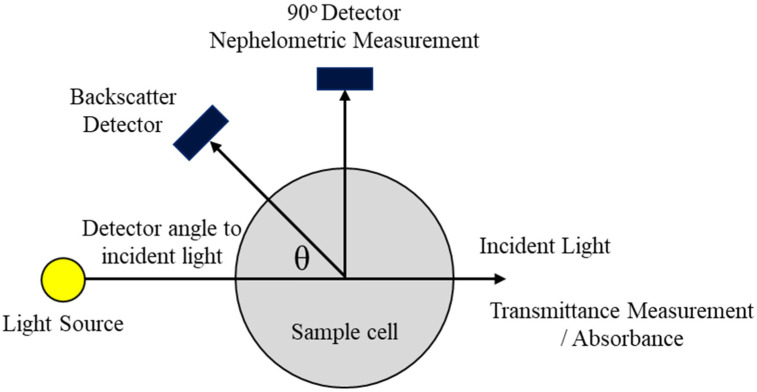
Turbidity Measuring Techniques.

There are two international standards for turbidity, namely ISO 7027 and USEPA 180.1, mostly using Kaolin or Formazine as standard samples. The turbidity can be measured with transmission-light attenuation, which uses FAU (Formazine Attenuation Unit) as a unit; or with scattering-light intensity, which uses FNU (Formazine Nephelometric Unit) as a unit [8]. In practice, most turbidity measurement equipment are scattering light-based, which use NTU (Nephelometric Turbidity Unit) as a display unit, for the sake of convenience. Besides these measurement techniques, backscattering refers to the measurement of scattered light at an angle between 90° to 180°. Figure 1 shows various configurations for measuring turbidity through an optical system [9].

With online water-quality analytical techniques having become ever more sophisticated, development of compact online monitoring devices is essential for IoT applications, instead of the traditional turbidity measurements involving on-site water sample collection and subsequent laboratory analysis, which is both labor intensive and costly [10,11]. New light sources have emerged in the form of light emitting diodes (LEDs) at considerably lower cost and higher reliability than conventional sources [12,13]. Tsao (2013) produced a turbidity-detecting chip with microelectric process technology, capable of making precision measurement for 0–125 NTU environmental water samples [14]. Wang et al. (2018) employed a laboratory-developed optical module capable of measurement with transmission and 90-degree scattering light at the same time, with precision reaching 0.1 NTU in the scope of 0–200 NTU but a 20% error in 200–1000 NTU [15]. Zhang and Chen (2017) put forth water-quality turbidity equipment featuring LED and anti-interference background light design, with a detection error < 0.5% in 0–120 NTU [16]. Parra et al. (2018) developed a smart turbidity optical sending module featuring a light-dependent resistor (LDR) and photodiode design for application in fishpond and marine environments, successfully detecting various kinds of algae [17,18,19,20]. Hakim et al. (2019) using SEN0189, a tested and characterized sensor module, analyzed this water turbidity sensor using an infrared LED and infrared phototransistor placed at 90° from the light source to detect a number of suspended solids in the water [21].

The studies above show that the development of compact real-time monitoring equipment combining increasingly mature optical technology and modular design has become a trend [22,23,24,25,26]. Online water quality monitoring can improve the treatment process with the real-time assessment of both source and treated water quality, and identification of contaminants and control of the treatment process [27,28,29]. Accordingly, this study develops a new monitoring module comprising a multi-lighting source design and diversified computing algorithms that consider not just scattering and transmission methods but also take reference into account for accuracy. This new design, which is under patent application, allowed multiple field samples measuring in various concentrations that can attain the goal of automated real-time water-quality monitoring.

## 2. Materials and Methods

### 2.1. Turbidity Determination Method

The study employs the HACH 2100P turbidity meter with precision and resolution of ±2% and 0.01 NTU, respectively, in measurements ranging from 0–1000 NTU, which meet international standards.

### 2.2. Optical Characteristics Measurement Equipment

Turbidity’s optical characteristics are established with a DH-2000 deuterium lamp (210–2500 nm, Ocean Optics) and USB2000, along with a spectrometer (200–1100 nm, Ocean Optics) and standard operating software to carry out the experiment.

Molds with different optical paths are produced via 3D printing and those with one-, three-, and five-centimeter optical paths are tested, with their pictures shown below (Table 1):

### 2.3. Sources of Samples for Turbidity Analytical Experiment

The study conducted various turbidity experiments including standard turbidity solution, tap water, and environmental samples. Standard turbidity solution was prepared by the certified reference materials (Cat. 246149, Formazin Turbidity Standard, 4000 NTU, Hach Company, Loveland, CO, USA) and then adjusted with deionized water to obtain desired concentrations of turbidity. Tap water and environmental samples were sampled from the Industrial Technology Research Institute and Toucian River, respectively; with sample sources and base concentration scope shown in Table 2.

### 2.4. Establishment of Turbidity Predication Model

A complete turbidity predication model was developed by utilizing standard turbidity solution to define absorption or scattering of wavelength, followed by the establishment of an algorithm via the statistical method. The unit turbidity’s optical contribution was then modified to establish the algorithm application according to the specific fields. The major steps are as follows:

1. Establishment of turbidity optical characteristics

Conduct qualitative analysis on standard turbidity solution, scanning its absorbed and scattering optical data for establishment of characteristic wavelength.

2. Establishment of turbidity prediction model

Establish turbidity prediction model by coupling turbidity characteristics wavelength, results from qualitative analysis with standard turbidity solution, and standard turbidity solution in various concentrations, to estimate the turbidity of a real-world water sample.

3. Estimate of actual turbidity concentration

To verify the reliability of an established prediction model, the actual sample concentration was compared against the estimated sample concentration to see whether the average error falls within the scope of expected values. The prediction model must be revised until the average error is less than 20%.

### 2.5. Turbidity Optical Experiment Plan

After securing turbidity optical characteristics with aforementioned optical equipment, conduct tests on various optical paths and scattering/absorption signals. New turbidity sensing modules were designed based on testing results, and finally, the established turbidity prediction model with the results of tap water and environmental water samples was verified. The experimental plan is shown in Table 3 below:

### 2.6. Statistical Analysis

All field water samples were measured by both the standard method (HACH 2100P) and study-design method. All experimental data in this study were obtained as average value after triplicated measurement. The measurement data were calculated and compared by statistical analysis with the mean difference tested against a population mean = 0 with an F-test (*p* = 0.01) [30].

## 3. Results and Discussion

### 3.1. Results of Turbidity Optical Characteristics

**Figure 2:**
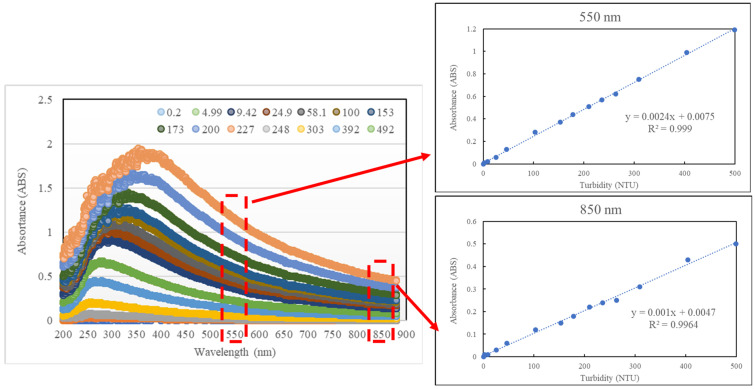
Absorbance of standard turbidity solutions in concentrations ranging 200–890 nm.

Figure 2 shows testing results of optical equipment with absorbance in turbidity ranging in wavelength 200–890 nm, according to which the relationship between UV (200–380 nm), VIS (380–750 nm) and NIR (>750 nm) and the turbidity coefficient of determination (R^2^) stand at 0.876–0.988, 0.985–0.999, and 0.996–0.998, respectively. The results show that visible light and near infrared light have a higher correlation with absorbance of standard turbidity solutions, exhibiting sufficient discriminatory ability with turbidity ranging from 0–500 NTU, according to the spectrum. Using international standards ISO 7027 and USEPA 180.1 as references, the study selected 550 and 850 nm wavelengths as the light sources in the subsequent design of sensing equipment. It should provide a concise and precise description of the experimental results and their interpretation, as well as the experimental conclusions that can be drawn.

### 3.2. Testing Results for Different Optical Paths

Establish low and high turbidity calibration curves, at 0–100 and 0–500 NTU, respectively, with 1-, 3-, and 5-cm optical-path molds and wavelengths at 550 (VIS) and 850 (NIR) nm. Experimental results are shown in Table 4, according to which with a 1 cm optical path under VIS conditions, the lowest detection limits for low and high turbidity stand at 6.25 and 5.56 NTU, respectively, compared with 20 and 14.29 NTU under NIR conditions. With a 3 cm optical path under VIS conditions, the lowest detection limits for low and high turbidity amount to 1.26 and 1.61 NTU, respectively, compared with 3.1 and 3.57 NTU under NIR conditions. With a 5 cm optical path under VIS conditions, the lowest detection limits for low and high turbidity reach 0.88 and 1.35 NTU, respectively, compared with 2.11 and 2.56 NTU under NIR conditions.

The experimental results exhibit that a 1 cm optical path mold has a higher lowest detection limit, indicating less sensitivity for low turbidity, although its discriminatory ability for high turbidity is still good. Therefore, it is more suitable for measuring a high-turbidity water body. In contrast, a 5 cm optical path mold has a good correlation and lower detection limit for low turbidity but sharp decline in correlation for a high-turbidity water sample, indicating that it is more suitable for measuring a low-turbidity water body. Meanwhile, 3 cm optical path mold performs well in correlation and lowest detection two aforementioned molds. As water treatment plants and rivers are two possible application fields, this study conducts further tests and subsequently establishes algorithms for 3 cm optical molds.

### 3.3. New Turbidity Sensing Device Design and Function Test

#### 3.3.1. Preliminary Test of Prototype Module Functions

**Figure 3:**
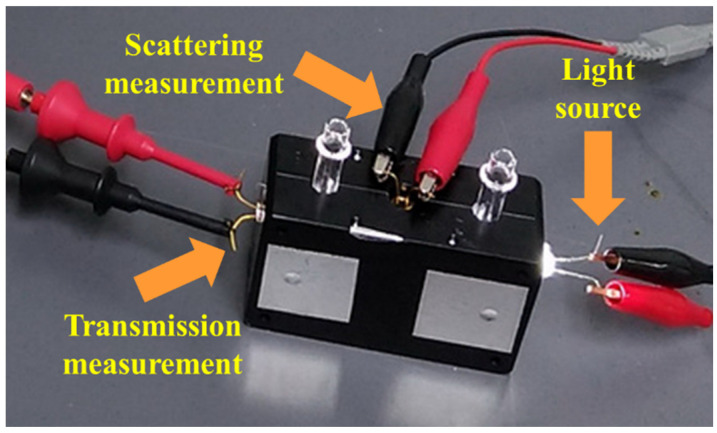
Prototype experimental mold of turbidity sensing module.

Utilize the aforementioned testing results to design a turbidity sensing module prototype (Figure 3), with incidence light being 550 and 850 nm LED, for functional tests on “scattering strength” and absorbance,” with results as follows:

1. Testing of turbidity sensing module for scattering signals

**Figure 4:**
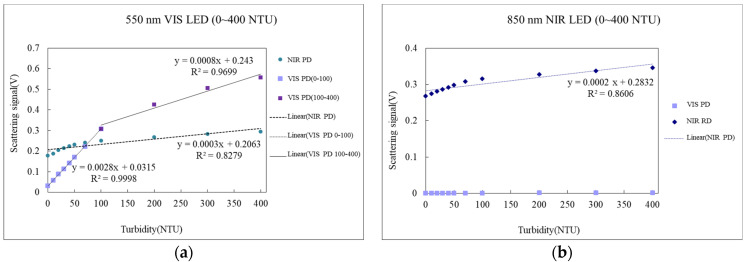
Results of testing of turbidity sensing modules for scattering signals. (**a**) 550 nm LED. (**b**) 850 nm LED.

Testing results of the aforementioned turbidity sensing module for two scattering signals, namely 550 and 850 nm LED, are shown in Figure 4, according to which there is good linear results for a 550 nm LED scattering signal within the concentration scope of 0–50 NTU under both VIS or NIR (IR or NIR). There, however, exhibit two different information sources within the concentration scope of 50–400 NTU, one being the difference in linear slope within the concentration scope from that in low concentration; the other being a lower linear effect within the scope than low concentration, although the PD result in both cases is consistent. Testing for 850 nm LED under NIR exhibit similar results but VIS PD does not respond to 850 nm scattering light, which, thus, is subsequently removed.

2. Testing of turbidity sensing module for absorbance

**Figure 5:**
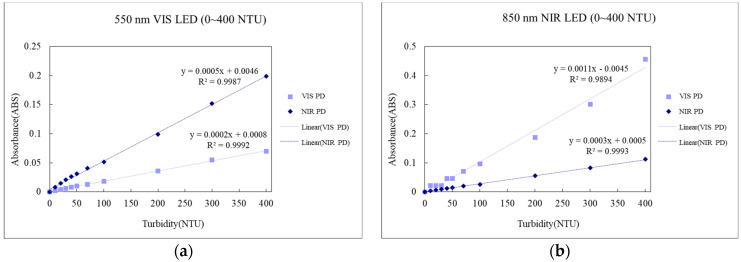
Results of testing of turbidity sensing modules for absorbance. (**a**) 550 nm LED. (**b**) 850 nm LED.

Testing results of the aforementioned turbidity sensing module for absorbance at 550 and 850 nm LED, are shown in Figure 5, according to which there is good linear results for 550 nm LED absorbance within the concentration scope of 0–400 NTU under both VIS or NIR, with the outcome for 850 nm LED remaining the same.

#### 3.3.2. Establishment of Algorithm for Turbidity Sensing Modules

Based on the aforementioned results concerning turbidity optical characteristics, the team establishes a turbidity prediction module for estimating the water sample’s turbidity. Five algorithm models for the turbidity prediction module were established based on measurement results from scattering light intensity, degree of transmission and reference lighting-source intensity with every lighting source. The five algorithm models were described as follows:

1. Pure scattering model (Ts 1): Turbidity computation with scattering-light intensity.
2. Scattering/reference specific value model (Ts 2): Turbidity computation with scattering light intensity after modification according to reference light intensity.
3. Scattering/transmitting specific value model (Ts 3): Turbidity computation with scattering light intensity after modification according to transmitting light intensity.
4. Pure transmission model (Tt 1): Turbidity computation with variation of transmitting light intensity, according to Beer-Lambert Law.
5. Transmitting/reference specific value model (Tt 2): Turbidity computation with variation of transmitting light intensity after modification according to reference light intensity, according to Beer-Lambert Law.

#### 3.3.3. New Turbidity Sensing Module Design

**Figure 6:**
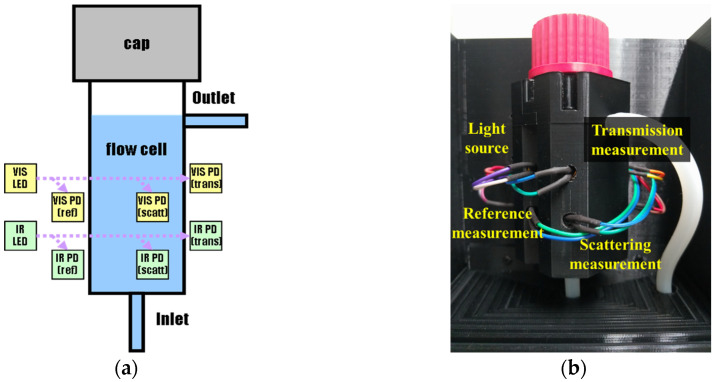
Turbidity sensing module (**a**) Design graph. (**b**) Optical module.

At present, two internationally accepted turbidity regulatory standards, USEPA 180.1 and ISO 7027, are based on scattering at 90 degrees, with visible light (400–600 nm) and near infrared light (850–870 nm) as mainstream lighting sources, coupled with various sensing components for determination at different concentrations. Based on the international norms and aforementioned testing results, the study designed a new turbidity sensing module, as shown in Figure 6a, which features multi-light detectors and twin lighting sources, with visible LED light applicable to transparent water body and near infrared LED light applicable to colored water body. Each lighting source possesses three sets of light detectors (scattering light + transmission light + reference light), which, coupled with the aforementioned five measurement models, enable users to select an optimal measurement model for different turbidity scope.

Modules are made of black photophobic materials, capable of avoiding the interference of light-ray scattering. The optical influence factor is included in the design of the sample’s cuvette, such as light transmitting axis in parallel, usage of quartz glass for the convenience of replacement and cleaning, or a bubble chamber in the tank to avoid the refraction interference of the water sample’s bubbles.

The optical design of this study was established from both the optimal testing results for different optical paths (Section 3.2) and specification of current commercial quartz glass. Basic design numbers of the resulting “new turbidity sensing module”, as shown in Figure 6b, are as follows:

∗ Incidence lighting source: 550 & 850 nm LED
∗ Sensor: VIS & NIR & Reference PD
∗ Transmitting optical path: 2.5 cm
∗ The measurement position of scattering intensity: 1.25 cm from optical center axis

### 3.4. Verification of the Algorithms of Turbidity Sensing Modules

After establishment, verify the turbidity sensing modules and five algorithms with standard turbidity solution and environmental water sample, for comparison with water-sample standard values with the HACH 2100P turbidity meter, with the verification results shown as follows:

#### 3.4.1. Results of Establishment of Standard Turbidity Solutions

As shown in Table 5, in the case of VIS lighting sources, three models of two optical signals, namely Ts2, Ts3, and Tt2, generate good estimate results in the range of 0–50 NTU, but estimate results of only scattering light and transmission light intensity tend to cause larger errors. Another finding is that the estimate error of the scattering-light intensity model will increase, along with the increase in concentration. Usage of three scattering light intensify models Ts1, Ts2, and Ts3 in the range of 50–400 NTU generate a larger error in estimate results, while estimate results for transmitting light intensify models Tt1 and Tt2 yield a smaller error, with Tt2, which also takes reference light intensity into account, boasting the smallest error. Verification results of the application of the five algorithm models in standard turbidity solution with different concentrations in the case of NIR lighting source are about the same as the aforementioned VIS lighting source, as shown in Table 6. Given the aforementioned verification results, the study employs a “low-concentration scattering” prediction model, coupled with a “high-concentration transmitting” model, for turbidity sensing modules, in principle, along with usage of scattering light coupled with reference and transmitting light intensity models Ts2 and Ts3 for verification in the case of low concentration (0–50 NTU), or reference light intensity model Tt2 for verification in the case of medium and high concentration (50–400 NTU).

#### 3.4.2. Verification Results for Field Water Sample

**Figure 7:**
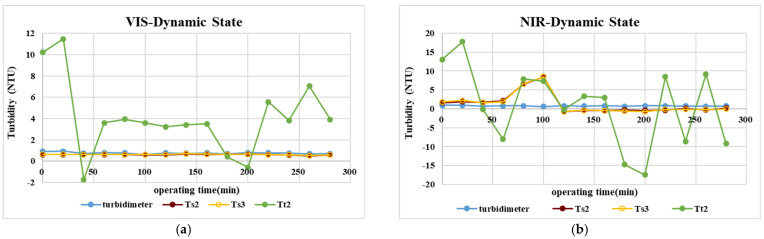
Continuing monitoring results of tap water turbidity verified by different algorithm models. (**a**)VIS. (**b**)NIR.

Figure 7 shows the turbidity estimate result with tap water as continuous influent using the aforementioned calibration curve for standard turbidity solutions, according to which estimate results of prediction models Ts2 and Ts3 are closer to actual measurement results (Ts2: F = 3.47, *p* > 0.01; Ts3: F = 3.69, *p* > 0.01), compared with a larger difference in Tt2, a finding verified by the average error and percentages of average error, as shown in Table 7 and Table 8. The outcome is perhaps due to the lower contribution of unit turbidity to degree of optical transmission, leading to lower sensitivity and stability of Tt2 in low turbidity (<1.0 NTU), as a result of which the algorithm is not suited to tap-water measurement. In the case of VIS LED lighting source, the average error of estimate results stands at around 20%, a far cry from over 200% for NIR LED, possibly due to the longer wavelength of NIR LED, which tends to cause diffraction dampening light quantity and thus unstable performance in top water with lower turbidity.

**Figure 8:**
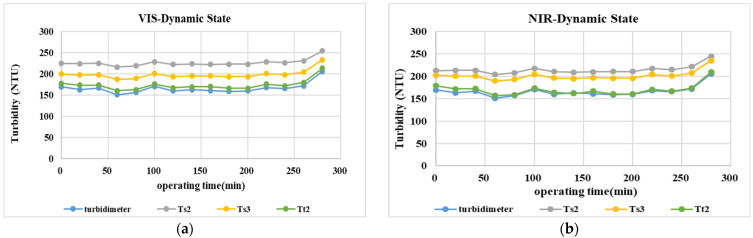
Continuing monitoring results for stream water turbidity verified by different lighting sources. (**a**) VIS measurement. (**b**) NIR measurement.

Figure 8 shows the turbidity estimate result with Toucian River water as continuous influent using the aforementioned calibration curve for standard turbidity solutions, according to which estimate results of prediction models Ts2 and Ts3 have a higher difference from actual measurement results, compared with Tt2 with results closer to actual measurement results (F = 1.02, *p* > 0.01), a finding verified by the average error and percentages of average error, as shown in Table 9 and Table 10, contrary to the outcome in the case of tap water entirely. The result is perhaps due to a more scattering situation caused by particles in other water samples with an increase of turbidity, in sharp contrast to good estimate results in the case of low turbidity.

Error of estimate results of the Tt2 algorithm model stand at 2.3% in the case of the NIR LED lighting source and 4.6% in the VIS LED lighting source, much lower than a 10% error of common commercial instruments, showing the model is suitable for estimating turbidity water samples with relatively high concentrations with both lighting sources.

Results from the application of the developed turbidity monitoring devices, along with five algorithm models, show that it needs more than two kinds of lighting sources, such as scattering light/reference light, to attain accurate monitoring, rather than just scattering light or transmission light alone.

Meanwhile, in the comparison of low and high concentrations, it is suggested to make an estimate with a proper inflection point, coupled with scattering and transmission algorithm models, to meet the need for environmental monitoring with higher variation in concentration and enable multi-field applications.

## 4. Conclusions and Suggestions

### 4.1. Conclusions

1. For new turbidity sensing modules based on optical principles, verification results show that
  (1) Prediction models Ts2 and Ts3 featuring VIS/LED/scattering are suggested to be used for monitoring tap water or low-concentration water samples (0–50 NTU), with a relative error < 20%.
  (2) Prediction model Tt2 featuring NIR LED/absorbance is suggested for use in monitoring high-concentration water samples (50–400 NTU), with a relative error < 3%; given the situation of overall measurement scope, the optimal option is based on the principles of “low-concentration scattering” coupled with “high-concentration transmission,” using VIS LED as a lighting source and models Ts2 and Ts3 for low concentration (0–50 NTU) and Tt2 for high concentration (50–400 NTU).
2. The new modularized online turbidity monitoring device developed by the team boasts multiple lighting sources/multiple parameters, which, coupled with self-developed algorithm models, is applicable to various fields with flexible change of monitoring models, possessing high commercialization potential.

### 4.2. Suggestion

Given susceptibility of optical sensors to pollution leading to overestimates of monitored water quality, we suggest developing a systematic cleaning model for module design in the future, which, coupled with completed design for reference light, can expand the application scope of optical sensors.

## Figures and Tables

**Table 1:** Photos of molds with various optical paths.

Optical Path: 1 cm	Optical Path: 3 cm	Optical Path: 5 cm
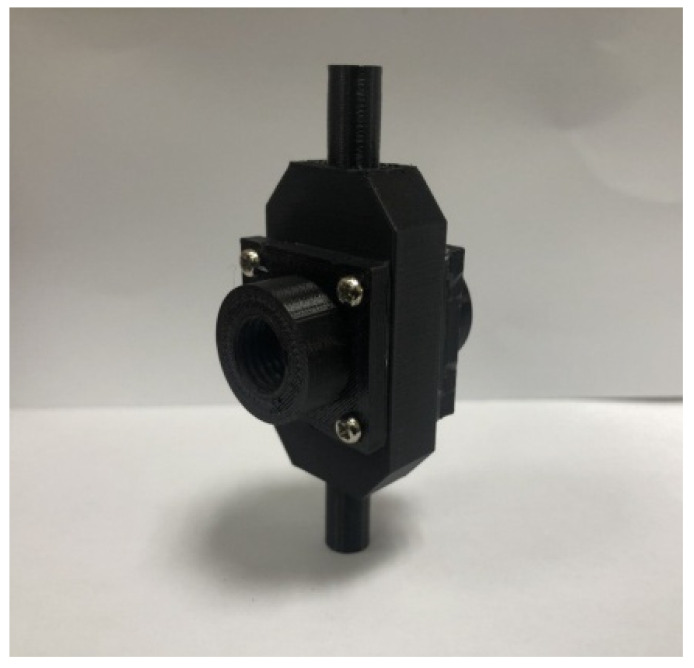	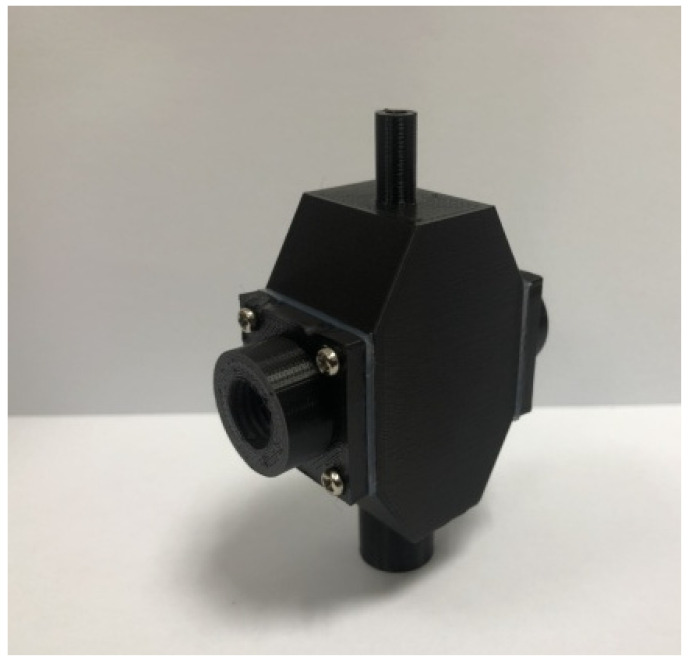	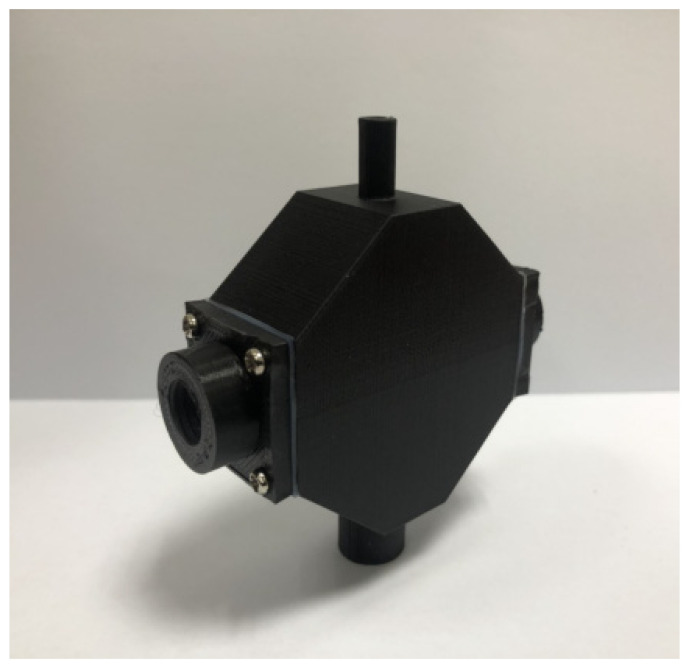

**Table 2:** Sample sources and basic concentrations.

Sample	Source	Concentration Scope (NTU)
Standard turbidity solution	HACH Formazin Turbidity Standard	0–4000
Tap water	Tap water from Industrial Technology Research Institute Kuangfu premises	0.2–0.5
Environmental water sample	Toucian River and silt	20–500

**Table 3:** Turbidity optical experiment plan.

Experimental Plan				
Item	Experiment Title	Experiment Sample	Instrument in Use	Experimental Conditions
1.	Turbidity optical characteristics	Standard turbidity solution	USB 2000+ Spectrum	200–890 nm
2.	Test on different optical paths	Standard turbidity solution	USB 2000+ Spectrum	1/3/5 cm
3.	Turbidity sensing module —Scattering signal testing	Standard turbidity solution	Prototype experimental mold for turbidity sensing	Three-centimeter transmitting optical path 1.5-cm transmitting optical path 1.5-cm scattering-light distance from lighting source
4.	Turbidity sensing module—absorbance testing	Standard turbidity solution	Prototype experimental mold for turbidity sensing	
5.	Verification of turbidity algorithm model	Tap water Environmental water	Prototype turbidity sensing module	Dynamic

**Table 4:** Various optical path molds’ calibration curves and lowest detection limits for wavelengths 550 nm and 850 nm.

Optical Path	Wavelength	Calibration Curve Scope	R^2^	Lowest Detection Limit (NTU)
1 cm	550 nm	0–100 NTU	0.934	6.25
		0–500 NTU	0.991	5.56
	850 nm	0–100 NTU	0.886	20
		0–500 NTU	0.991	14.29
3 cm	550 nm	0–100 NTU	0.995	1.26
		0–500 NTU	0.983	1.61
	850 nm	0–100 NTU	0.992	3.1
		0–500 NTU	0.996	3.57
5 cm	550 nm	0–100 NTU	0.988	0.88
		0–500 NTU	0.940	1.35
	850 nm	0–100 NTU	0.985	2.11
		0–500 NTU	0.993	2.56

**Table 5:** Estimate results for concentrations of standard turbidity solutions (VIS).

HACH 2100P	Predictive Value (NTU)					Percentage Error (%)				
Standard Value (NTU)	Ts1	Ts2	Ts3	Tt1	Tt2	Ts1	Ts2	Ts3	Tt1	Tt2
0.64	1.92	0.73	0.75	0.66	0.74	200.5	13.6	17.2	2.7	15.4
6.08	7.31	5.96	5.93	7.09	5.94	20.2	2.0	2.5	16.6	2.3
11.8	11.7	11.7	11.7	11.1	11.9	0.6	0.7	0.6	6.0	0.9
20.4	25.0	25.0	24.8	20.7	21.3	22.6	22.5	21.3	1.5	4.3
32.7	34.9	35.0	34.8	33.3	32.4	6.8	6.9	6.5	1.8	0.8
44.6	40.4	40.4	40.7	44.1	44.5	9.3	9.4	8.7	1.1	0.2
52.3	49.6	49.6	48.5	50.5	51.2	5.1	5.1	7.3	3.4	2.1
97.6	155.0	153.5	126.8	105.0	103.9	58.8	57.2	30.0	7.6	6.5
183.0	253.2	249.9	220.0	209.7	200.0	38.4	36.6	20.2	14.6	9.3
218	249.9	249.3	232.0	220.1	216.5	14.6	14.4	6.4	1.0	0.7
303	289.1	290.4	303.5	311.0	310.9	4.6	4.2	0.2	2.7	2.6
338	296.6	297.0	317.7	327.5	330.2	12.3	12.1	6.0	3.1	2.3
407	341.9	343.5	380.4	386.6	397.1	16.0	15.6	6.5	5.0	2.4
Average						32.8%	15.4%	10.6%	5.2%	3.9%

**Table 6:** Estimate results for concentrations of standard turbidity solutions (NIR).

HACH 2100P	Predictive Value (NTU)					Percentage Error (%)				
Standard Value (NTU)	Ts1	Ts2	Ts3	Tt1	Tt2	Ts1	Ts2	Ts3	Tt1	Tt2
0.64	0.12	0.08	0.09	-0.64	0.78	81.9	86.8	86.1	200.2	22.0
6.08	7.06	6.96	6.95	11.21	5.89	16.1	14.5	14.4	84.3	3.2
11.8	11.5	11.4	11.5	6.0	10.7	2.6	3.3	2.9	49.2	8.9
20.4	23.5	23.7	23.4	23.6	17.8	15.0	16.4	14.8	15.8	12.8
32.7	33.8	33.8	33.8	35.1	34.5	3.3	3.4	3.5	7.5	5.4
44.6	42.5	42.3	42.5	41.2	43.7	4.6	5.1	4.7	7.7	1.9
52.3	43.7	45.0	46.7	60.5	56.4	16.4	13.9	10.6	15.8	7.9
97.6	139.4	139.0	125.8	95.9	96.0	42.8	42.5	28.9	1.7	1.6
183	239.7	238.6	220.9	183.3	187.6	31.0	30.4	20.7	0.2	2.5
218	242.2	241.9	231.7	208.0	207.2	11.1	11.0	6.3	4.6	5.0
303	298.6	299.2	305.3	315.0	313.8	1.4	1.3	0.7	4.0	3.6
338	307.0	306.8	316.9	332.2	334.1	9.2	9.2	6.2	1.7	1.1
407	355.9	357.2	379.1	409.6	408.2	12.6	12.2	6.9	0.6	0.3
Average						19.6%	19.8%	16.7%	32.7%	6.3%

**Table 7:** Statistics of average error for continuing monitoring of tap water turbidity verified by different lighting sources.

Average Error (NTU)	Ts2	Ts3	Tt2
VIS	0.16	0.14	3.88
NIR	1.81	1.88	8.49

**Table 8:** Statistics of average error percentages for continuing monitoring of tap water turbidity verified by different lighting sources.

Average Error Percentages (%)	Ts2	Ts3	Tt2
VIS	19.6%	17.8%	487.8%
NIR	248.1%	257.2%	1093.2%

**Table 9:** Statistics of average error for continuing monitoring of stream water turbidity verified by different lighting sources.

Average Error (NTU)	Ts2	Ts3	Tt2
VIS	60.23	32.73	7.62
NIR	48.44	34.99	3.86

**Table 10:** Statistics of average error percentages for continuing monitoring of stream water turbidity verified by different lighting sources.

Error Percentage (%)	Ts2	Ts3	Tt2
VIS	36.5%	19.8%	4.6%
NIR	29.4%	21.2%	2.3%

## Data Availability

The data presented in this study are available on request from the corresponding author. The data are not publicly available because this research was funded by the government project.
